# Diacylglycerol Kinases (DGKs): Novel Targets for Improving T Cell Activity in Cancer

**DOI:** 10.3389/fcell.2016.00108

**Published:** 2016-10-17

**Authors:** Matthew J. Riese, Edmund K. Moon, Bryon D. Johnson, Steven M. Albelda

**Affiliations:** ^1^Division of Hematology/Oncology, Department of Medicine, Medical College of WisconsinMilwaukee, WI, USA; ^2^Blood Center of Wisconsin, Blood Research InstituteMilwaukee, WI, USA; ^3^Division of Pulmonary, Allergy, and Critical Care, Department of Medicine, University of PennsylvaniaPhiladelphia, PA, USA; ^4^Division of Hematology/Oncology/Transplant, Department of Pediatrics, Medical College of WisconsinMilwaukee, WI, USA

**Keywords:** diacylglycerol, diacylglycerol kinase, immunotherapy, CD8+ T cell, T cell receptor

## Abstract

Diacylglycerol kinases (DGKs) are a family of enzymes that catalyze the metabolism of diacylglycerol (DAG). Two isoforms of DGK, DGKα, and DGKζ, specifically regulate the pool of DAG that is generated as a second messenger after stimulation of the T cell receptor (TCR). Deletion of either isoform in mouse models results in T cells bearing a hyperresponsive phenotype and enhanced T cell activity against malignancy. Whereas, DGKζ appears to be the dominant isoform in T cells, rationale exists for targeting both isoforms individually or coordinately. Additional work is needed to rigorously identify the molecular changes that result from deletion of DGKs in order to understand how DAG contributes to T cell activation, the effect of DGK inhibition in human T cells, and to rationally develop combined immunotherapeutic strategies that target DGKs.

## Diacylglycerol kinases in T cell receptor signal transduction

Diacylglycerol kinases (DGKs) represent a family of enzymes that catalyze phosphorylation of the membrane lipid sn-1,2 diacylglycerol (DAG) to form phosphatidic acid (PA) (Eichmann and Lass, 2015). In T cells, DAG is formed downstream of the T cell receptor (TCR) after activation of the gamma 1 isoform of phospholipase C (PLCγ1) and cleavage of phosphatidylinositol 4,5-biphosphate (PIP_2_) into DAG and an additional second messenger, inositol 1,4,5-triphosphate (IP_3_) (Krishna and Zhong, 2013). Whereas, IP_3_ is important in facilitating release of calcium from the endoplasmic reticulum, DAG interacts with other proteins important in TCR signal transduction, such as Protein kinase C (predominantly θ isoform in T cells, but also isoforms ε and η; Quann et al., 2011) and the Ras activating protein RasGRP1 (Krishna and Zhong, 2013). Biochemically, targeting the activity of DGKs in T cells, either by germline deletion, or with chemical inhibitors, results in enhanced and sustained signaling downstream of T cells, as assessed by prolonged phosphorylation of downstream molecules, such as extracellular signal-related kinases 1/2 (ERK1/2; Zhong et al., 2003; Olenchock et al., 2006; Riese et al., 2011). Although, three isoforms of DGK are known to be present within T cells (DGKα, DGKδ, and DGKζ), only two, DGKα and DGKζ, are thought to play an important role in facilitating DAG metabolism downstream of the TCR (Joshi and Koretzky, 2013). The function of DGKδ is unknown in T cells; its role in facilitating metabolic flexibility between lipid and carbohydrate utilization suggest that it may regulate pools of DAG unrelated to TCR signaling (Chibalin et al., 2008). The signaling changes resulting from the absence of DGKα or DGKζ alter the transcriptional program of activated T cells. For instance, generation of the transcription factor AP1, which is dependent on Ras/ERK signaling, is decreased in stimulated Jurkat T cells overexpressing DGKζ (Zhong et al., 2002). Similarly, NF-κB (nuclear factor kappa-ligh-chain-enhancer of activated B cells), a critical transcription factor activated downstream of PKCθ in T cells, is present at increased levels after stimulation of DGKζ-deficient lymphocytes as compared to DGKζ-replete cells (Schmidt et al., 2013), though other data suggests that the regulation of NF-κB may, in some instances, be positively regulated by DGKs (Yang et al., 2016).

The change in activation of transcription factors in stimulated T cells after manipulation of DGK activity correlates with changes in T cell activation markers and function. For instance, overexpression of DGKα or DGKζ in Jurkat T cells results in decreased expression of the activation marker CD69 after stimulation through the TCR complex (Sanjuán et al., 2001; Zhong et al., 2002), and overexpression of DGKα induces a state of decreased functional activity resembling an anergy-like state (Zha et al., 2006). In contrast, deletion of DGKα or DGKζ results in T cells with enhanced production of effector cytokines, such as IL2 and IFNγ, and enhanced proliferation (Zhong et al., 2003; Olenchock et al., 2006). Inhibition of DGKα also allows T cells to overcome TCR signaling defects present in human X-linked lymphoproliferative disease (XLP-1) resulting from the loss of SAP [signaling lymphocytic activation molecule (SLAM)-associated protein (Baldanzi et al., 2011; Ruffo et al., 2016)], along with uncontrolled effector T cell expansion after exposure to Epstein Barr virus (EBV) characteristic of this disease (Ruffo et al., 2016).

## Targeting diacylglycerol kinases to enhance T cell anti-tumor activity

Given the enhanced functional activity conferred by loss of DGKs in T cells, our group and others have tested the hypothesis that these proteins may serve as useful targets for enhancing T cell anti-tumor activity. Recently, strategies to target negative regulators of T cells to enhance their anti-tumor activity have been successfully translated from basic science studies into clinical care (Byrne et al., 2015; Sharma and Allison, 2015; Shin and Ribas, 2015; Callahan et al., 2016). Although, antibodies directed against CTLA-4 and PD-1 are the most prominent examples of therapies that have generated clinical responses in human malignancy, there is significant interest in identifying additional inhibitory regulators of T cells to combine with existing approaches and to use in instances where blockade of PD-1 and other immune checkpoints is ineffective (Restifo et al., 2016). We focused our studies on DGKζ, since that enzyme appears to represent the dominant isoform in T cells, based on a direct comparison of TCR signal strength between T cells deficient in either DGKα or DGKζ (Joshi et al., 2013). Using an EL4-ova subcutaneous model system to permit tracking of immune responses, we observed that DGKζ^−/−^ mice had an increased frequency of tumor rejection, along with a trend toward increased number of tumor-specific CD8^+^ T cells (Riese et al., 2011). Additionally, we demonstrated that adoptively transferred naïve (Riese et al., 2011) or activated (Riese et al., 2013) tumor-specific effector T cells displayed increased activation by tumor and resultant inhibition of tumor growth. While these studies relied on strong-antigen driven tumor models, it is likely that loss of DGKs also enhances T cell anti-tumor activity in tumors with low-grade antigens, since DAG-mediated activation of RasGRP1 regulates the threshold for T cell activation (Das et al., 2009), and earlier studies implicated a role for Ras in Jurkat T cell activation mediated by low grade TCR stimulus (Perez de Castro et al., 2004).

DGKα has also been evaluated as a potential target to improve T cell activity against tumor, based on the observation that DGKα is upregulated in certain inhibited T cell conditions, such as anergy (Zha et al., 2006), and that DGKα is upregulated in tumor-infiltrating lymphocytes in human renal cell carcinoma (Prinz et al., 2012). For instance, our own study using adoptive transfer of CAR (chimeric antigen receptor)-T cells demonstrated similar increases in efficacy (compared to wild type T cells) between T cells deficient in DGKα or DGKζ in the treatment of murine mesothelioma (Riese et al., 2013). Additionally, a study testing the importance of DGKα in glioblastoma multiforme (GBM) cells found that concurrent administration of the relatively non-specific DGKα inhibitor R59022 resulted in decreased growth of intracranially injected GBM tumors. Although, a preponderance of evidence suggested that the decreased tumor growth in this model resulted from inhibition of DGKα within the tumor cells, modulation of immune activity was not assessed, and could have been contributory (Dominguez et al., 2013). DGKs also play a role in limiting the activity of NK cells isolated from tumors in patients with renal cell carcinoma, since the addition of either IL-2 or DGK inhibitor (R59022) to culture media improves the impaired function of tumor-associated NK cells (Prinz et al., 2014). Recently, a more specific inhibitor for DGKα has been developed that may be useful to extend these studies into additional tumor models (Liu et al., 2016). Although a direct comparison has not to-date been performed comparing tumor growth in DGKα^−/−^ and DGKζ^−/−^ mice, future studies will undoubtedly provide additional comparisons between the two genotypes with respect to T cell anti-tumor immunity.

## Mechanism of enhanced T cell anti-tumor activity in DGKζ-deficient T cells

**Figure 1:**
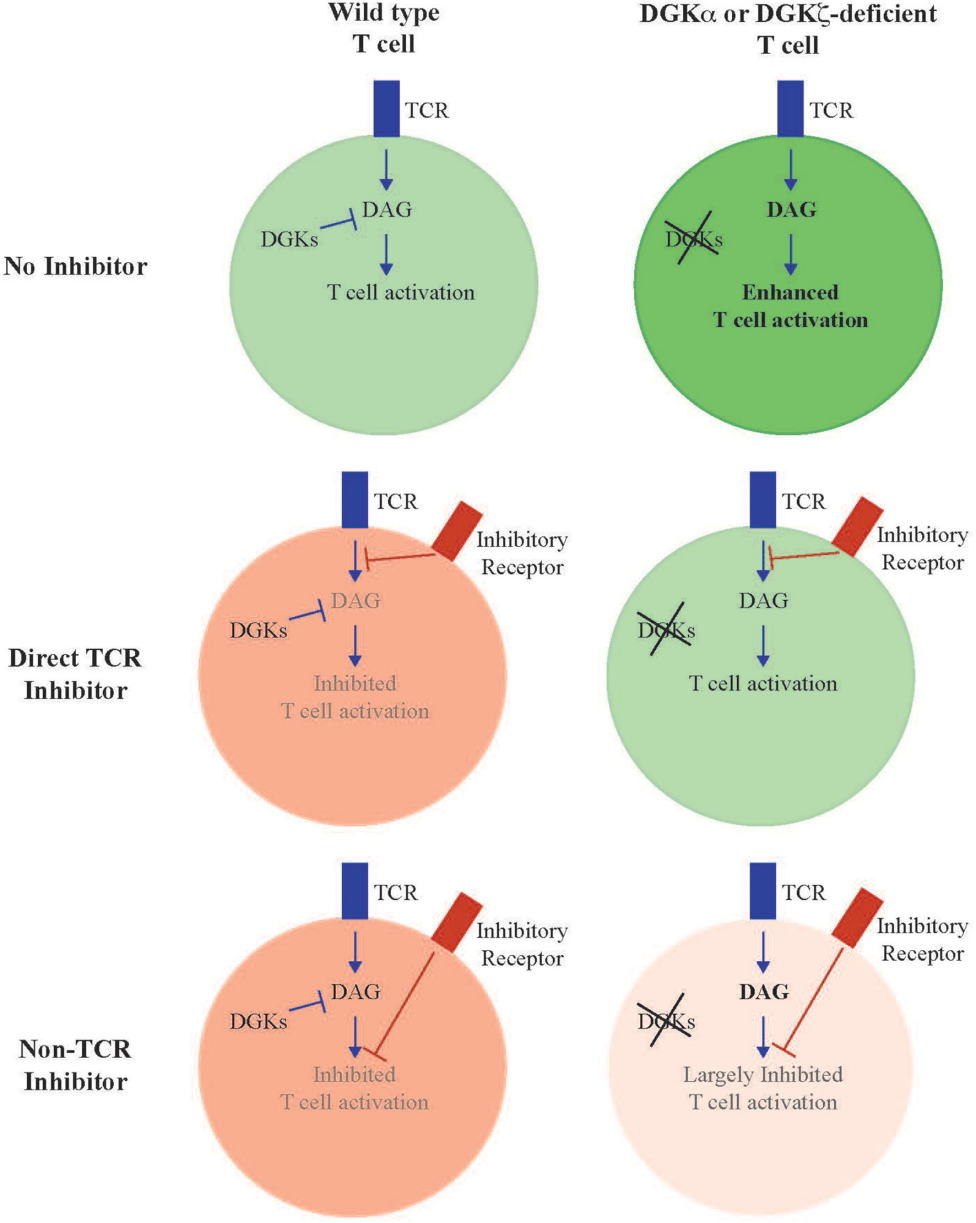
**Model of enhanced T cell activity in DGK-deficient T cells**. DGKα and DGKζ function to metabolize DAG generated downstream of TCR activation. Deletion of either or both isoforms results in T cells with enhanced TCR signal transduction downstream of DAG and resultant enhanced effector functions (“No inhibitor”). Loss of DGK confers resistance to inhibitory pathways that function through direct interference of TCR signal transduction, since these inhibitory events occur proximal to generation of DAG (“Direct TCR Inhibitor”). We predict that T cells deficient in DGKs remain sensitive to inhibitory pathways that mediate their effect independent of interference with TCR signal transduction (“Non-TCR Inhibitor”), or for pathways that inhibit TCR signaling at rate limiting steps downstream of DAG formation.

Enhanced anti-tumor activity observed in DGKζ-deficient T cells was initially thought to result from increased cytokine production generated after TCR stimulation; however, it is clear that intrinsic insensitivity to inhibitory signals in the tumor microenvironment is also an important determinant. In broad terms, T cell inhibitory factors can be broadly separated into two groups: those that inhibit T cells by directly inhibiting proximal TCR signal transduction, and those that inhibit T cells independent of attenuation of TCR signaling. Examples of immunosuppressant pathways that facilitate direct inhibition of TCR signaling include PD-1 (Chemnitz et al., 2004; Parry et al., 2005; Yokosuka et al., 2012), Lag3 (Okazaki et al., 2011), Prostglandin E_2_ (PGE_2_) (Wehbi and Taskén, 2016), adenosine 2A receptor (Linnemann et al., 2009; Linden and Cekic, 2012), and as we have recently identified, TGFβ (Arumugam et al., 2015; Newman et al., 2016). Whereas, PD-1 and TGFβ inhibit TCR signaling by directly or indirectly recruiting inhibitory tyrosine phosphatases, such as SHP-1 or SHP-2 (Src homology region 2 domain-containing phosphatase-1/2) to the cell surface, adenosine 2A, and PGE_2_ receptors activate protein kinase A (PKA) leading to Csk-mediated inhibition of the proximal activating tyrosine kinase Lck (Newick et al., 2016). In contrast, Lag3 appears to oppose TCR signaling via a KIEELE motif that acts through an unclear mechanism (Freeman and Sharpe, 2012). In either case, these inhibitory factors act to oppose activation events facilitated by proximal tyrosine protein kinases, such as Lck or Zap-70, that are responsible for initiating TCR signal cascades and are upstream of DAG generation. In contrast are inhibitory factors that act predominantly by inhibiting T cells independent of TCR activation and DAG generation. These include a subset of immune checkpoint receptors, such as Tim3 (Jones et al., 2008; Lee et al., 2011), and CTLA-4. Although some reports have suggested direct proximal inhibition of TCR mediated by CTLA-4 (Lee et al., 1998), CTLA-4 more likely functions predominantly as a sink to sequester CD80 and CD86, the ligands of the co-stimulatory molecule CD28 (Green et al., 1994; Walunas et al., 1996; van der Merwe et al., 1997; Collins et al., 2002). T cells deficient in DGKs may demonstrate differential sensitivity to inhibition mediated by dependently or independently of TCR signaling (Figure 1, Table 1). For instance, a test of numerous T cell inhibitory pathways revealed that, in contrast to wild type T cells, T cells lacking DGKζ demonstrate reduced inhibition of IFNγ production in the presence of high concentrations of TGFβ, Prostaglandin E_2_ (PGE_2_) or adenosine (Riese et al., 2013). This suggests that one can predict, in broad terms, how DGK-deficient T cells will respond to various immunosuppressive pathways within the tumor microenvironment, in that one would anticipate DGK-deficient T cells to be insensitive to inhibitors that directly attenuate TCR signaling, such as PD-1 or Lag3, but sensitive to inhibitory pathways that do not directly interfere with TCR signaling, such as Tim3 or CTLA-4. This model also predicts which immune targets might be optimally paired with agents that block DGKs, such that one would anticipate little synergy with direct regulators of TCR signaling, but potential excellent synergy with TCR-independent inhibitors. Experiments are currently ongoing to test these hypotheses.

**Table 1:** **Relative insensitivity to inhibitory stimuli of CD8+ T cells**.

**Inhibitory stimuli/receptor**	**Inhibitor-mediated fold ↓ in IFNγ by CD8+ T cell**		**Does inhibitory pathway directly impact TCR signaling?**	**References**
	**WT**	**DGKζ^−/−^**		
TGFβ	35x	1x	Yes	Arumugam et al., 2015
PGE2	2x	1.5x	Yes	Riese et al., 2013
Adenosine	2x	1.2x	Yes	Riese et al., 2013
PD-1	4x	Unknown	Yes	Parry et al., 2005
Lag3	2x	Unknown	Yes	Hannier et al., 1998; Huang et al., 2015
Tim-3	None	Unknown	No	Lee et al., 2011
CTLA-4	2x	Unknown	Uncertain	Krummel and Allison, 1995; Walunas and Bluestone, 1998

## Potential issues arising from targeting DGKs

The diverse set of cellular functions governed by DAG and PA, including signal transduction (Mérida et al., 2008), lipid biogenesis (Shulga et al., 2013), and membrane trafficking (Cho and Stahelin, 2005), presents challenges for broadly targeting DGK activity therapeutically, especially since the enzymatic specificity of the 10 mammalian DGKs is highly conserved, with the exception of DGKε (Jennings et al., 2015). Thus, isolation of a compound with specificity toward an individual isoform may prove difficult, a problem exacerbated by the paucity of structural information available about eukaryotic DGKs. Although, a prokaryotic form of DGK has been solved structurally (Li et al., 2013), it is sufficiently divergent from eukaryotic isoforms that it provides little value in predicting active site topography of eukaryotic DGKs. For instance, prokaryotic DGKA is not limited to enzymatic activity against lipids, but can also catalyze reactions using glycerol and water as substrates for phosphorylation (Ullrich et al., 2011). Therefore, approaches to target non-catalytic domains of DGKζ may provide the best means to achieve isoform specificity. The structural domains of DGKζ have been well defined, and include a C1 domain, a MARCKS domain, an ankyrin repeat domain, and a C-terminal PDZ-binding domain (Joshi and Koretzky, 2013). Of these domains, only the MARCKS domain and ankyrin repeat domain are unique to DGKζ among the 10 DGK family members (Joshi and Koretzky, 2013). Furthermore, only the MARCKS domain, a substrate for serine/threonine phosphorylation by PKCα (Topham et al., 1998), is required for DGKζ function (Santos et al., 2002). Thus, targeting the MARCKS domain may be an effective strategy for therapeutic targeting of DGKζ, in a manner that confers specificity.

Apart from concerns with isotype specificity, therapeutic targeting of DGKα and DGKζ could result in deleterious “on-target” effects, such as enhanced cellular proliferation and autoimmunity. As expected, mice with deletions of both DGKα and DGKζ generate T cells with enhanced TCR signaling downstream of DAG, as well as more potent effector functions after *in vitro* stimulation relative to single knockouts or wild type T cells (Guo et al., 2008; Riese et al., 2013). However, the activated level of Ras/Erk signal transduction in double knockout mice (DKO) results in thymic lymphomagenesis (Guo et al., 2008). This pro-malignant potential of enhanced DAG signaling is consistent with data from human patients with T cell acute lymphoblastic leukemia (T-ALL), in which RasGRP1, the Ras activating protein activated by DAG, has been found to be frequently overexpressed (Hartzell et al., 2013). Thus, caution must be used if DGK germline deletion (e.g., using CRISPR-based approaches) is used as a means to target DGKs in adoptive T cell therapies. Generating auto-immune disease may also be an issue when targeting DGKs. Although mice deficient in DGKα, DGKζ, or both do not develop overt autoimmunity, it is likely that the mice would develop enhanced T cell responses in autoimmune models such as experimental autoimmune encephalitis (EAE), especially given the role DGKs play in limiting activation of Mnk1/2-mediated activation and development of encephalitis in the EAE model (Gorentla et al., 2013). A possible explanation for the lack of overt autoimmunity in DGKζ or DKO mice may be the concurrent enhanced generation of natural T regs in these mice (Joshi et al., 2013), although this remains speculative, and raises the additional consideration that thymic development of T cells may be impacted by manipulation of DGK activity. For instance, constitutive expression of a membrane-bound form of DGKα leads to accumulation of immature CD8+ “single positive” T cells within the thymus in addition to peripheral lymphopenia (Almena et al., 2013), and DKO mice demonstrate a severe impairment in thymic development of invariant NKT cells, a subset of innate T cells (Shen et al., 2011). Ongoing experiments are needed to test whether DGK-deficient mice will develop more severe disease than wild type counterparts after autoimmune challenges, and to determine whether subtle alterations are present in the thymic development of other conventional T cell subsets.

## Conclusions

As immunotherapeutic approaches come to the forefront of cancer treatment, there is an increased need to evaluate proteins and molecules that inhibit the immune system, especially in T cells. Diacylglycerol kinases should warrant a high degree of consideration in these targeting strategies, ideally with the development of small molecule inhibitors. In the meanwhile, it may be advantageous to move forward with gene manipulation in adoptive cellular therapies. The ability to very specifically target DGKs within only the transferred T cells will minimize systemic side effects. It will also be possible to include suicide genes to enable destruction of the transferred T cells, should it become necessary. A better understanding of the changes that result from acute and long-term targeting of DGKs should help discern the effectiveness of this strategy both alone, and in combination with other therapies designed to induce immune cell activation.

## Author contributions

All authors contributed to the conception and design of the manuscript and contributed writing to the paper.

## Funding

Funding provided by NIH K08 CA151893 (MR), NIH K08 CA163941 (EM), American Cancer Society (MR), Kathy Duffey Fogerty Family Foundation (MR), HRHM Program of MACC Fund (BJ).

### Conflict of interest statement

The authors declare that the research was conducted in the absence of any commercial or financial relationships that could be construed as a potential conflict of interest. MR and SA are co-holders of a pending patent application on targeting DGKs for adoptive cellular therapies.
